# Developing Strategy to Predict the Results of Prostate Multiparametric Magnetic Resonance Imaging and Reduce Unnecessary Multiparametric Magnetic Resonance Imaging Scan

**DOI:** 10.3389/fonc.2021.732027

**Published:** 2021-09-14

**Authors:** Junxiao Liu, Shuanbao Yu, Biao Dong, Guodong Hong, Jin Tao, Yafeng Fan, Zhaowei Zhu, Zhiyu Wang, Xuepei Zhang

**Affiliations:** ^1^Department of Urology, The First Affiliated Hospital of Zhengzhou University, Zhengzhou, China; ^2^Key Laboratory of Precision Diagnosis and Treatment for Chronic Kidney Disease in Henan Province, Zhengzhou, China

**Keywords:** prostate cancer, magnetic resonance imaging, prostate-specific antigen, prostate-specific antigen density, multivariate model

## Abstract

**Purpose:**

The clinical utility of multiparametric magnetic resonance imaging (mpMRI) for the detection and localization of prostate cancer (PCa) has been evaluated and validated. However, the implementation of mpMRI into the clinical practice remains some burden of cost and availability for patients and society. We aimed to predict the results of prostate mpMRI using the clinical parameters and multivariable model to reduce unnecessary mpMRI scans.

**Methods:**

We retrospectively identified 784 men who underwent mpMRI scans and subsequent prostate biopsy between 2016 and 2020 according to the inclusion criterion. The cohort was split into a training cohort of 548 (70%) patients and a validation cohort of 236 (30%) patients. Clinical parameters including age, prostate-specific antigen (PSA) derivates, and prostate volume (PV) were assessed as the predictors of mpMRI results. The mpMRI results were divided into groups according to the reports: “negative”, “equivocal”, and “suspicious” for the presence of PCa.

**Results:**

Univariate analysis showed that the total PSA (tPSA), free PSA (fPSA), PV, and PSA density (PSAD) were significant predictors for suspicious mpMRI (*P* < 0.05). The PSAD (AUC = 0.77) and tPSA (AUC = 0.74) outperformed fPSA (AUC = 0.68) and PV (AUC = 0.62) in the prediction of the mpMRI results. The multivariate model (AUC = 0.80) had a similar diagnostic accuracy with PSAD (*P* = 0.108), while higher than tPSA (*P* = 0.024) in predicting the mpMRI results. The multivariate model illustrated a better calibration and substantial improvement in the decision curve analysis (DCA) at a threshold above 20%. Using the PSAD with a 0.13 ng/ml^2^ cut-off could spare the number of mpMRI scans by 20%, keeping a 90% sensitivity in the prediction of suspicious MRI-PCa and missing three (3/73, 4%) clinically significant PCa cases. At the same sensitivity level, the multivariate model with a 32% cut-off could spare the number of mpMRI scans by 27%, missing only one (1/73, 1%) clinically significant PCa case.

**Conclusion:**

Our multivariate model could reduce the number of unnecessary mpMRI scans without comprising the diagnostic ability of clinically significant PCa. Further prospective validation is required.

## Introduction

Prostate cancer (PCa) is the second most common malignancy in men, with over 1 million new cases and 375,304 deaths in 2020 (1). The diagnostic tools of PCa mainly includes digital rectal examination (DRE), prostate-specific antigen (PSA) test, multiparametric magnetic resonance imaging (mpMRI), and prostate biopsy (2). DRE requires extensive experience, and has a limited value in decision-making (3). PSA is a better predictor of PCa than DRE, and is the gold standard for PCa screening (4). The mpMRI has a good sensitivity for the detection and localization of clinically significant PCa (CSPCa, defined as Gleason score ≥ 3 + 4) (5, 6). Prostate biopsy is the gold standard for PCa diagnosis, but is invasive.

While these risk stratification tools have an additional value in the diagnostic pathway of PCa, it is controversial to perform mpMRI and prostate biopsy in every man with an elevated serum tPSA level and/or other clinical suspicion, in consideration of the costs for patients, the burden, and availability for society. Performing mpMRI and/or prostate biopsy among men with a high risk of CSPCa could be an acceptable option (7). A dozen of risk calculators incorporating clinical variables and/or novel biomarkers have been developed to predict the results of prostate biopsy and to reduce unnecessary biopsy by 36%–66% (8–12). However, the knowledge about developing a strategy to predict the results of mpMRI and select patients who could benefit from mpMRI is limited (13, 14).

Our prior study, consistent with other studies, found that clinical parameters such as age, PSA derivates [total PSA (tPSA), free/total PSA (f/tPSA), and PSA density (PSAD)], and prostate volume (PV) were significant predictors for PCa and CSPCa (8, 15). Therefore, we question whether the negative and equivocal mpMRI scans could be limited using a model based on these clinical parameters among men with an elevated PSA level. Our study aimed to predict the results of mpMRI using the inexpensive, inexperience, and readily available clinical parameters and multivariable model, and to assess the impact of potentially avoidable mpMRI scans. Overall, this study will be helpful for optimizing the diagnostic pathway, implementing a precision treatment strategy, and reducing the burden for patients and health care providers.

## Materials and Methods

### Study Populations

This retrospective study was approved by the institutional review board. We identified 903 consecutive patients who underwent PSA test, mpMRI scans, and subsequent prostate biopsy between April 2016 and March 2020 at our medical center (**Supplementary Figure 1**). Patients were excluded due to incomplete data (94 cases) or being diagnosed with other types of tumor/cancer (25 cases), leaving 784 (87%) patients available for analysis (**Supplementary Figure 1**). The 70% and 30% of the study population were randomly divided into a training cohort (548 cases) and a validation cohort (236 cases), respectively (**Supplementary Figure 1**).

### Clinical, Imaging, and Pathological Parameters Collection

The clinical parameters including age at prostate biopsy, serum tPSA and fPSA values, PV, and reports of mpMRI examination were extracted from clinical records. The serum tPSA and fPSA were measured by immunofluorescence assay. PV was measured by mpMRI examination using the 3.0-T MRI system (SIEMENS, Germany). The protocol of mpMRI examination complied with the guidelines of the European Society of Urology Radiology, and included T2-weighted Imaging (T2WI), diffusion-weighted imaging (DWI), and dynamic contrast-enhanced imaging (DCE). The prostate mpMRI images were interpreted by two experienced radiologists with at least three years of prostate mpMRI experience. The mpMRI results were divided into three groups: “negative”, “equivocal”, and “suspicious” for the presence of PCa, according to the mpMRI reports. The “negative”, “equivocal”, and “suspicious” for MRI-PCa corresponded to the PI-RADS 1 or 2, PI-RADS 3, and PI-RADS 4 or 5 according to the latest Prostate Imaging Reporting and Data System version 2 (PI-RADS v2) guideline (16).

### Prostate Biopsy and Histopathological Diagnosis

All patients underwent a transrectal ultrasound (TRUS)-guided systematic 12-point prostate biopsy (15). If there are suspected malignant nodules by mpMRI and/or ultrasound, additional 1–5 needles were performed in regions with cognitive MRI-TRUS fusion and/or abnormal ultrasound echoes. Biopsy cores were analyzed according to the standards of the ISUP (17).

### Statistical Analysis

We described the profile of age, PSA derivates (tPSA, fPSA, f/tPSA, PSAD), PV, and prostate biopsy results of the enrolled patients by the category of mpMRI results. The χ^2^ test or Fisher’s exact test was used to analyze categorical data. The Mann-Whitney U test was used to analyze ranked data. Student’s t-test or ANOVA was used to analyze continuous data. Multivariable logistic regression analysis with a stepwise strategy was used to develop models to predict mpMRI results. The area under the ROC curve (AUC) was used to evaluate the diagnostic accuracy of the clinical parameters and multivariable model. Differences between the AUCs were compared using the method of DeLong et al. (18). The calibration plot was used to assess the performance characteristics of the models. Calibration was assessed by grouping men in the validation cohort into delices (each of size 23 or 24), and then comparing the mean of the predicated probabilities and the observed proportions. The sum squares of the residues (SSR) were used to assess the deviation of calibration plots from the 45° line (19). Decision-curve analysis was used to measure the clinical utility. All tests were two sided with significance level set at 0.05. Data cleaning and analyses were conducted using the R statistical software (Version 4.0.2).

## Results

A total of 784 patients underwent PSA test, mpMRI scans, and subsequent prostate biopsy enrolled in this study. Of the enrolled patients, 296 (37.8%) were negative for MRI-PCa, 133 (17.0%) were equivocal for MRI-PCa, and 355 (45.2%) were suspicious for MRI-PCa (**Table 1**). The clinical parameters including age, tPSA, fPSA, f/tPSA, PSAD, PV, and prostate biopsy categorized by the mpMRI results are displayed in **Table 1**. The training and validation cohorts consisted of 548 (70%) and 236 (30%) patients. The clinical parameters were similar between the training cohorts and validation cohorts (each *P* > 0.05, **Supplementary Table 1**). Of the validation cohorts, 91 (39%) were negative for MRI-PCa, 38 (16%) were equivocal for MRI-PCa, 107 (45%) were suspicious for MRI-PCa; 138 (58%) were benign biopsy, 12 (5%) were PCa (GS = 3 + 3), and 86 (36%) were CSPCa (**Supplementary Table 1**).

**Table 1:** The clinical parameters and biopsy results by category of mpMRI results between April 2016 and March 2020.

Clinical parameters	mpMRI examination			
	Negative (n = 296)	Equivocal (n = 133)	Suspicious (n = 355)	*P*
Age (years)	67 (62–72)	68 (61-75)	68 (63–74)	0.032
tPSA (ng/ml)	11.5 (7.71–18.3)	12.7 (5.98–22.3)	23.2 (9.71–45.3)	<0.001
fPSA	1.65 (0.96–2.58)	1.49 (0.84–3.02)	2.59 (1.21–5.31)	<0.001
f/tPSA	0.14 (0.10–0.20)	0.14 (0.10–0.20)	0.11 (0.07–0.19)	0.002
PSAD (ng/ml^2^)	0.21 (0.13–0.34)	0.22 (0.11–0.40)	0.48 (0.21–0.96)	<0.001
PV (ml)	58 (37–84)	51 (34–74)	46 (33–68)	<0.001
Biopsy result, No. (%)				<0.001
No-PCa	254 (86)	99 (74)	104 (29)	
GS = 3 + 3	14 (5)	9 (7)	23 (6)	
GS = 3 + 4	12 (4)	10 (8)	28 (8)	
GS = 4 + 3	9 (3)	4 (3)	75 (21)	
GS ≥ 8	7 (2)	11 (8)	125 (36)	

tPSA, total prostate-specific antigen; fPSA, free PSA; f/tPSA, free PSA/total PSA; PSAD, PSA density; PV, prostate volume; GS, Gleason score.

### Univariate Analysis of Clinical Parameters for Suspicious MRI-PCa

In the univariate analysis, all clinical parameters except age and f/tPSA were significant predictors for suspicious MRI-PCa (each *P* < 0.05, **Table 2**). The risk of suspicious MRI-PCa increased with tPSA (OR = 1.03, 95% CI: 1.02–1.04), fPSA (OR = 1.14, 95% CI: 1.07–1.21), and PSAD (OR = 3.03, 95% CI: 2.05–4.49), but it was conversely associated with PV (OR = 0.995, 95%CI: 0.990–0.999) (**Table 2**). PSAD (AUC = 0.77) had a higher diagnostic accuracy compared with fPSA (AUC = 0.68, *P* = 0.017) and PV (AUC = 0.62, *P* < 0.001) (**Table 2**), and showed a similar diagnostic accuracy with tPSA (AUC = 0.74, *P* = 0.144) in the prediction of MRI-PCa (**Table 2**).

**Table 2:** Univariate and multivariate regression analysis of clinical parameters to predict suspicious MRI-PCa in the validation cohort.

Clinicalparameters	Univariate analysis			Multivariate analysis		
	OR (95% CI)	AUC (95% CI)	*P*	Coefficient	OR (95% CI)	*P*
Intercept	NA		NA	-1.537	NA	0.019
Age (yrs)	1.01 (1.00–1.03)	0.59 (0.51–0.66)	0.135	NA	NA	NA
tPSA (ng/ml)	1.03 (1.02–1.04)	0.74 (0.68–0.81)	<0.001	0.031	1.03 (1.02–1.04)	<0.001
fPSA	1.14 (1.07–1.21)	0.68 (0.61–0.75)	<0.001	0.070	1.07 (1.00–1.14)	0.038
f/tPSA	1.52 (0.69–3.37)	0.61 (0.53–0.68)	0.302	NA	NA	NA
PV (ml)	0.995 (0.990–0.999)	0.62 (0.54–0.69)	0.017	-0.012	0.99 (0.98–0.99)	<0.001
PSAD (ng/ml^2^)	3.03 (2.05–4.49)	0.77 (0.71–0.83)	<0.001	NA	NA	NA

tPSA, total prostate-specific antigen; fPSA, free PSA; f/tPSA, free PSA/total PSA; PV, prostate volume; PSAD, PSA density; GS, Gleason score; NA, not applicable.

### Development of a Multivariate Model to Predict Suspicious MRI-PCa

**Figure 1:**
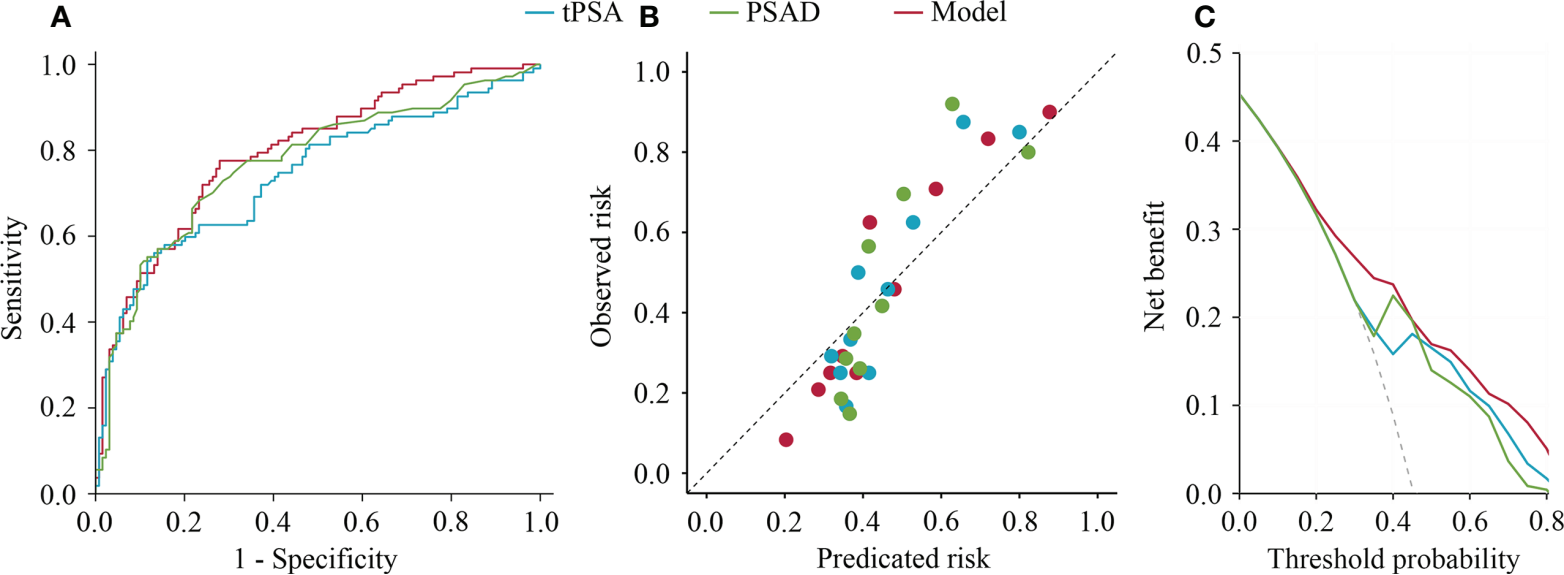
Receiver operating characteristics curves, calibration plot, and decision curve analysis of tPSA, PSAD, and multivariable model for predicting suspicious prostate cancer by mpMRI. **(A)** Receiver operating characteristics curves; **(B)** Calibration plot; **(C)** Decision curve analysis.

In the stepwise multivariate analysis, tPSA (*P* < 0.001), fPSA (*P* = 0.038), and PV (*P* < 0.001) remained in the multivariate model as significant predictors for suspicious MRI-PCa. The multivariate model (AUC = 0.80) outperformed tPSA (*P* = 0.024), and behaved similarly with PSAD (*P* = 0.108) in the prediction of suspicious MRI-PCa (**Table 2** and **Figure 1A**). Additionally, the calibration plot indicated an excellent concordance in the multivariate model (SSR = 0.118), followed by tPSA (SSR = 0.146), and PSAD (SSR = 0.241) (**Figure 1B**). The DCA showed that the multivariate model had the highest net clinical benefit across the threshold probabilities above 20% (**Figure 1C**). It was considered that the multivariate model was most helpful to rule out the “Suspicious MRI-PCa”.

### Impact of the Clinical Parameters and Multivariate Model on mpMRI Scans Reduced and CSPCa Diagnosis Delayed

To further assess the potential clinical benefit of the tPSA, PSAD, and multivariate model, the clinical consequences of using various cut-offs for the tPSA, PSAD, and multivariate model are listed in **Table 3**. Using of a 32% cutoff for the multivariate model would allow for reducing 64/236 (27%) mpMRI scans, while keeping 96/107 (90%) sensitivity in the prediction of suspicious MRI-PCa. At the same level of sensitivity as the multivariate model to predict suspicious MRI-PCa, applying the tPSA and PSAD could reduce 37/236 (16%) and 48/236 (20%) mpMRI scans, respectively. All the 236 patients in the validation cohort obtained clear pathological results of prostate biopsy. Assuming that the indications for subsequent biopsies were only based on the mpMRI findings, biopsies among men who would not have undergone mpMRI scans revealed three CSPCa using the tPSA, three CSPCa using the PSAD, and one CSPCa using the multivariate model in the validation cohort.

**Table 3:** The diagnostic performance of tPSA, PSAD, and multivariate model in prediction of suspicious MRI-PCa in the validation cohort.

Strategies	Sensitivity	Cut-off	mpMRI scans reduced (n = 236), n (%)	Suspicious mpMRI delayed		
				No-PCa (n = 28) n (%)	GS = 3 + 3 (n = 6), n (%)	GS ≥ 3 + 4 (n = 73), n (%)
tPSA	106/107 (99%)	0.90 ng/ml	3 (1)	0 (0)	0 (0)	1 (1)
PSAD	106/107 (99%)	0.03 ng/ml^2^	4 (2)	0 (0)	0 (0)	1 (1)
Multivariate	106/107 (99%)	0.25	19 (8)	1 (4)	0 (0)	0 (0)
tPSA	102/107 (95%)	4.50 ng/ml	19 (8)	2 (7)	0 (0)	3 (4)
PSAD	102/107 (95%)	0.10 ng/ml^2^	27 (11)	2 (7)	0 (0)	3 (4)
Multivariate	102/107 (95%)	0.29	41 (17)	4 (14)	0 (0)	1 (1)
tPSA	96/107 (90%)	6.70 ng/ml	37 (16)	8 (29)	0 (0)	3 (4)
PSAD	96/107 (90%)	0.13 ng/ml^2^	48 (20)	8 (29)	0 (0)	3 (4)
Multivariate	96/107 (90%)	0.32	64 (27)	10 (36)	0 (0)	1 (1)
tPSA	91/107 (85%)	9.10 ng/ml	65 (28)	10 (36)	1 (17)	5 (7)
PSAD	91/107 (85%)	0.18 ng/ml^2^	80 (34)	12 (43)	0 (0)	4 (5)
Multivariate	91/107 (85%)	0.35	85 (36)	14 (50)	0 (0)	2 (3)
tPSA	86/107 (80%)	10.7 ng/ml	89 (38)	11 (39)	1 (17)	9 (12%)
PSAD	87/107 (81%)	0.21 ng/ml^2^	92 (39)	15 (54)	0 (0)	5 (7)
Multivariate	86/107 (80%)	0.37	100 (42)	17 (61)	0 (0)	4 (5)

PCa, prostate cancer; CSPCa, clinically significant prostate cancer; GS, Gleason score; tPSA, total prostate-specific antigen; PV, prostate volume; SVI, seminal vesicle invasion; LNI, lymph node invasion.

## Discussion

The added value of mpMRI for the detection and localization of CSPCa has been validated (5, 12, 20). However, it is controversial to perform mpMRI in every man with an elevated serum tPSA level. Our study revealed that tPSA, fPSA, PV, and PSAD were significant predictors for suspicious MRI-PCa, and the number of mpMRI scans could be reduced based on the low cost and readily available clinical parameters. At the same level of sensitivity (90%) in the prediction of suspicious MRI-PCa, the multivariate model could reduce more mpMRI scans (27%) and missed less CSPCa (1%), compared with PSAD (20% and 4%) and tPSA (16% and 4%).

Reported proportions of the total negative MRI (PI-RADS 1-2) ranged from 37% to 58% for individual studies depending on the prevalence of PCa in the study populations (21–23). The ratios of negative MRI-PCa and equivocal MRI-PCa were 38% and 17% in our study. These indicate that the overuse of prostate mpMRI is common in the current healthcare environments, and it is essential to identify men who will benefit from mpMRI in the current MRI era. In this study, we assessed the inexpensive and readily available parameters as the predictor for suspicious MRI-PCa, and found that PSAD and tPSA had a higher diagnostic accuracy than other single parameters. However, the PV, which was used to calculate the PSAD and develop a multivariate model, were estimated by the mpMRI examination. However, the PV could be reliably measured by TRUS, which was a routine and low-cost procedure (24). Hence, an accurate PSAD could be obtained using TRUS before mpMRI without changing the clinical workflow.

To date, multivariate models or machine learning models for the detection of CSPCa have been developed in a growing body of literatures (8–12, 25). Studies demonstrated that a risk-based triage strategy could reduce more unnecessary biopsy and the overdiagnosis in comparison with single parameters (8, 11, 25). However, the study about developing a multivariate model to predict the results of prostate mpMRI and selecting patients who could benefit from mpMRI is limited (13, 14). The study by Alberts et al. introduced the concept of a patient triage strategy to avoid prostate mpMRI, and assessed the rate of potentially avoidable mpMRI by applying the risk calculators for detecting PCa (Rotterdam Prostate Cancer Risk Calculator, RPCRC) in a small cohort with one or more previously negative random TRUS-guided biopsies (14). The RPCRC (57/83, 69%) incorporating a multitude of variables spared less unnecessary mpMRI scans than our simple model (106/138, 77%) at the same level of sensitivity for the detection of CSPCa. These may indicate that commonly risk models for detecting PCa and/or CSPCa do not address the appropriate use of mpMRI. It is essential to establish risk models and decision thresholds for the prediction of mpMRI results.

In this study, our developed multivariate model including tPSA, fPSA, and PV has a similar diagnostic accuracy with the PSAD in the prediction of suspicious MRI-PCa (*P* = 0.108). This was consistent with the study by Dominik Deniffel (13). Although cross-study comparisons are challenging, our multivariate model (AUC = 0.80) performed similarly with the model developed by Dominik Deniffel (AUC = 0.75) in the prediction of mpMRI results (13). Using the two simple multivariate model could reduce above a quarter of mpMRI scans at a high sensitivity for the detection of CSPCa. It substantiates that the mpMRI scans could be reduced based on the readily available clinical parameters. The strength of our study was able to establish the definite link between mpMRI omission and the rate of CSPCa delayed. Some studies showed that a high-resolution micro-ultrasound had a comparable or higher sensitivity for the detection of CSPCa compared to mpMRI (26, 27), and was an independent parameter to predict the results of biopsy (28). In the further study, we will evaluate more convenient, low-cost, clinical parameters as predictors for the results of mpMRI, and to strengthen our multivariate model.

Our study was subject to several limitations. First, this study is a single center study based on a Chinese population, and limited by the inherent drawbacks of its retrospective design. The study results should be cautiously applied to other populations, and further prospective multicenter validation is required. Second, the PV used to calculate PSAD and build a multivariate model was estimated by mpMRI in our study. However, a study showed that PV could be reliably measured by TRUS (24), and a low-cost micro-ultrasound had a high sensitivity for the detection CSPCa (26, 27). Third, our model only included clinical parameters such as age, PSA test, and volume. The race, family history, and micro-ultrasound (26–28) will be considered in future studies to augment our multivariate model.

## Conclusions

Our study demonstrated that tPSA, fPSA, PV, and PSAD were significant predictors for the mpMRI results. The multivariate model based on the inexpensive and readily available clinical parameters could be used as an aid to select patients who could benefit from mpMRI and to reduce the unnecessary mpMRI scans without compromising the ability to diagnose CSPCa. Further prospective validation is required.

## Data Availability Statement

The raw data supporting the conclusions of this article will be made available by the authors, without undue reservation.

## Author Contributions

XZ, JL, and SY conceptualized, designed, and supervised the study. JL, SY, BD, and GH coordinated and participated in the data collection. SY and JL carried out the statistical analysis and drafted the manuscript. XZ, SY, JT, and FY provided guidance on the data analysis. JL, SY, ZZ, ZW, and XZ revised the manuscript. All authors contributed to the article and approved the submitted version.

## Funding

The research was supported by the Henan Medical Science and Technology Project [grant no. LHGJ20190181 (XZ) and LHGJ20200334 (SY)].

## Conflict of Interest

The authors declare that the research was conducted in the absence of any commercial or financial relationships that could be construed as a potential conflict of interest.

## Publisher’s Note

All claims expressed in this article are solely those of the authors and do not necessarily represent those of their affiliated organizations, or those of the publisher, the editors and the reviewers. Any product that may be evaluated in this article, or claim that may be made by its manufacturer, is not guaranteed or endorsed by the publisher.
