# Supplementary material for: Developing Strategy to Predict the Results of Prostate Multiparametric Magnetic Resonance Imaging and Reduce Unnecessary Multiparametric Magnetic Resonance Imaging Scan
Source: Front Oncol. 2021 Sep 14;11:732027. doi: 10.3389/fonc.2021.732027 (PMC8476778; doi:10.3389/fonc.2021.732027)
Supplement: Supplementary file 1 [file DataSheet_1.docx]

Supplementary Material

# Supplementary Figure 1


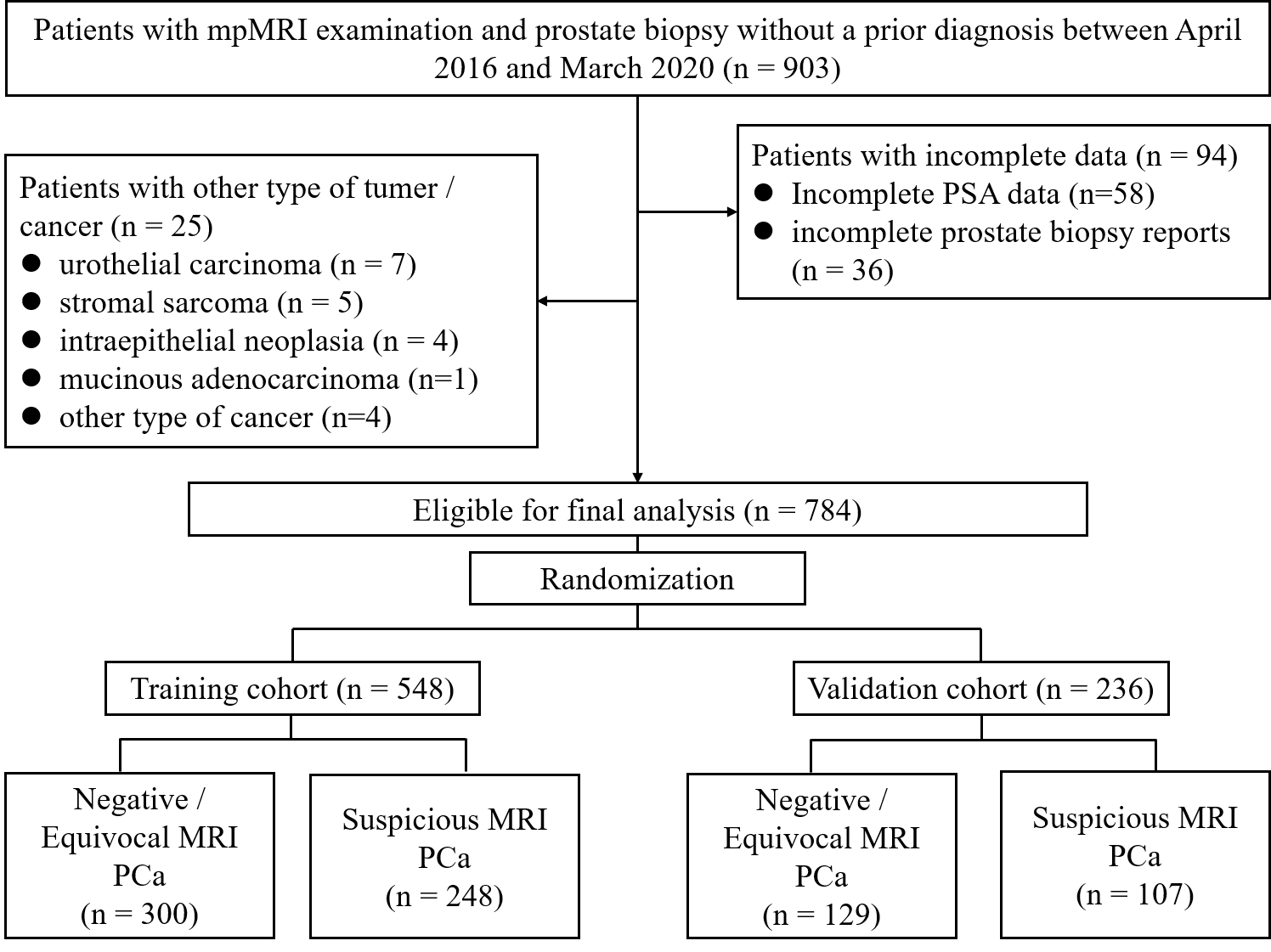


**Supplementary Figure 1.** Flowchart of study participants selection.

# Supplementary Table 1 | Comparison of clinical characteristics between training cohort, and validation cohort

| Clinical characteristics | Training cohort  (n=548) | Validation cohort  (n=236) | *P* |
| --- | --- | --- | --- |
| Age (years) | 68 (62-74) | 68 (62-73) | 0.294 |
| tPSA (ng/ml) | 14.8 (7.97-28.6) | 14.2 (8.82-29.5) | 0.600 |
| f/tPSA | 0.13 (0.09-0.20) | 0.13 (0.09-0.19) | 0.895 |
| PSAD (ng/ml^2^) | 0.28 (0.15-0.59) | 0.28 (0.15-0.63) | 0.898 |
| PV (ml) | 50 (34-73) | 53 (35-74) | 0.612 |
| MRI-PCa, No. (%) | |  | 0.902 |
| Negative | 205 (37) | 91 (39) |  |
| Equivocal | 95 (17) | 38 (16) |  |
| Suspicious | 248 (45) | 107 (45) |  |
| Biopsy result |  |  | 0.777 |
| No-PCa | 319 (58) | 138 (58) |  |
| GS≤3+3 | 34 (6) | 12 (5) |  |
| GS=3+4 | 38 (7) | 12 (5) |  |
| GS=4+3 | 61 (11) | 27 (11) |  |
| GS≥8 | 96 (18) | 47 (20) |  |

tPSA: total prostate-specific antigen; f/tPSA: free PSA / total PSA; PV: prostate volume; PCa: prostate cancer; GS: Gleason score.
